# Supplementary material for: Complexes of Glucarolactones with Water-Soluble Copolymers of N-Vinylpyrrolidone with N-Vinylamine as Inhibitors of β-Glucuronidase Efficacy
Source: Polymers (Basel). 2021 Dec 28;14(1):105. doi: 10.3390/polym14010105 (PMC8747385; doi:10.3390/polym14010105)
Supplement: Supplementary file 1 [file polymers-14-00105-s001.zip › polymers-1516876-supplementary .pdf]

polymers MDPI

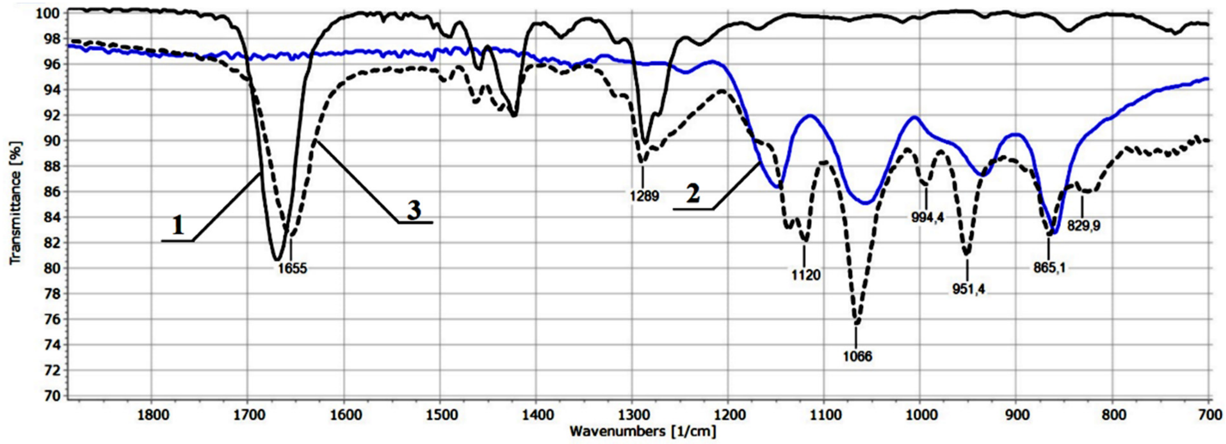

Figure 1. IR spectra of copolymer $[N\text{-}VP]_n\text{-}[N\text{-}BA]_m$ (1), equimolar mixture of Na_2HPO_4 and NaH_2PO_4 (2), mixture of the copolymer with sodium phosphates (3).

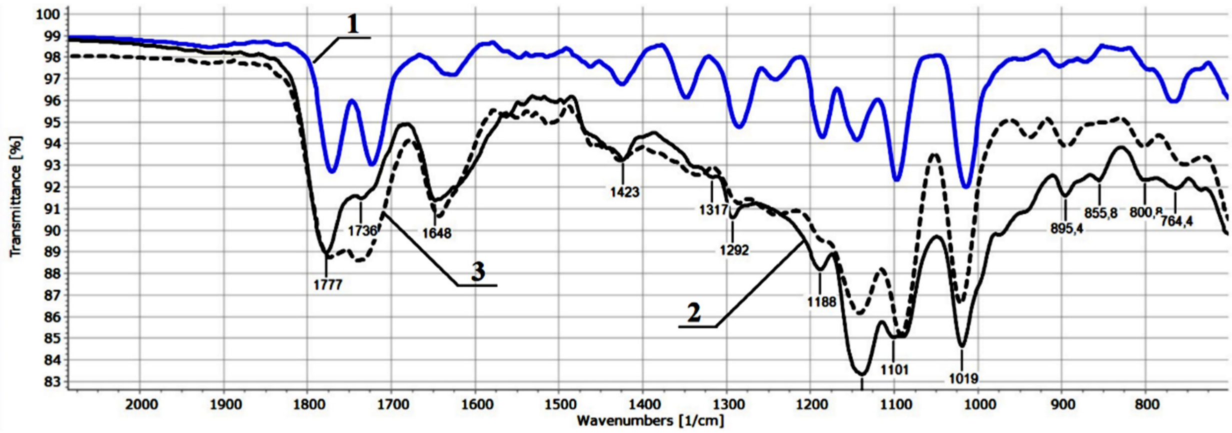

Figure 2. IR spectra of 1,4-GL (1), complex $[N\text{-}VP]_n\text{-}[N\text{-}VA]_m$ [1,4- and 6,3-GL] (2), Preparation without added sodium phosphates (3).
